# Production of Noncapped Genomic RNAs Is Critical to Sindbis Virus Disease and Pathogenicity

**DOI:** 10.1128/mBio.02675-20

**Published:** 2020-12-01

**Authors:** Autumn T. LaPointe, V Douglas Landers, Claire E. Westcott, Kevin J. Sokoloski

**Affiliations:** a Department of Microbiology and Immunology, School of Medicine, University of Louisville, Louisville, Kentucky, USA; b Center for Predictive Medicine and Emerging Infectious Diseases, University of Louisville, Louisville, Kentucky, USA; Indiana University Bloomington

**Keywords:** alphavirus, capping, RNA virus, nsP1, viral pathogenesis

## Abstract

Alphaviruses are positive-sense RNA viruses that utilize a 5′ cap structure to facilitate translation of viral proteins and to protect the viral RNA genome. Nonetheless, significant quantities of viral genomic RNAs that lack a canonical 5′ cap structure are produced during alphaviral replication and packaged into viral particles. However, the role/impact of the noncapped genomic RNA (ncgRNA) during alphaviral infection *in vivo* has yet to be characterized. To determine the importance of the ncgRNA *in vivo*, the previously described D355A and N376A nsP1 mutations, which increase or decrease nsP1 capping activity, respectively, were incorporated into the neurovirulent AR86 strain of Sindbis virus to enable characterization of the impact of altered capping efficiency in a murine model of infection. Mice infected with the N376A nsP1 mutant exhibited slightly decreased rates of mortality and delayed weight loss and neurological symptoms, although levels of inflammation in the brain were similar to those of wild-type infection. Although the D355A mutation resulted in decreased antiviral gene expression and increased resistance to interferon *in vitro*, mice infected with the D355A mutant showed significantly reduced mortality and morbidity compared to mice infected with wild-type virus. Interestingly, expression of proinflammatory cytokines was found to be significantly decreased in mice infected with the D355A mutant, suggesting that capping efficiency and the production of ncgRNA are vital to eliciting pathogenic levels of inflammation. Collectively, these data indicate that the ncgRNA have important roles during alphaviral infection and suggest a novel mechanism by which noncapped viral RNAs aid in viral pathogenesis.

## Abstract (precis)

Mosquito-transmitted alphaviruses have been the cause of widespread outbreaks of disease that can range from mild illness to lethal encephalitis or severe polyarthritis. There are currently no safe and effective vaccines or therapeutics with which to prevent or treat alphaviral disease, highlighting the need to better understand alphaviral pathogenesis to develop novel antiviral strategies. This report reveals production of noncapped genomic RNAs (ncgRNAs) to be a novel determinant of alphaviral virulence and offers insight into the importance of inflammation to pathogenesis. Taken together, the findings reported here suggest that the ncgRNAs contribute to alphaviral pathogenesis through the sensing of the ncgRNAs during alphaviral infection and are necessary for the development of severe disease.

## INTRODUCTION

Alphaviruses are positive-sense, single-stranded RNA arboviruses that are capable of causing severe disease. The natural enzootic transmission cycle of these viruses is between a mosquito vector and a mammalian host, typically rodents or birds, although epizootic spillover events can occur that result in infection of humans and equids. Alphaviruses are broadly categorized as either arthritogenic or encephalitic based on disease symptomology. The arthritogenic alphaviruses, such as Chikungunya virus (CHIKV) and Ross River virus (RRV), are capable of causing disease ranging from mild febrile illness to severe polyarthralgia, which can persist anywhere from weeks to years following infection (1–3). In contrast, the encephalitic alphaviruses, such as Venezuelan equine encephalitis virus (VEEV) and some strains of Sindbis virus (SINV), like the AR86 strain used in this study, can cause mild to severe neurological symptoms, including encephalitis that can potentially lead to the death of the host (3–5). While alphaviruses pose a large threat to public health, there are currently no safe and effective vaccines or antiviral therapies to prevent or treat alphaviral disease.

Alphaviruses produce three RNA species during infection: the genomic strand, which encodes the nonstructural proteins; the minus-strand RNA template; and the subgenomic RNA, which encodes the structural proteins. Both the genomic and subgenomic RNAs have a type 0 cap structure added to their 5′ ends to facilitate translation and protect the 5′ end of the transcripts (6–8). The addition of the cap structure to the 5′ end of viral RNAs (vRNAs) is primarily carried out by nonstructural proteins 1 and 2 (nsP1 and nsP2). nsP2 removes the 5′ γ-phosphate from the nascent vRNA molecule, while, in a separate reaction, the methyltransferase domain of nsP1 catalyzes the addition of a methyl group from S-adenosylmethionine to a GTP molecule, forming a covalent m7GMP-nsP1 intermediate (9, 10). The m7GMP moiety is then transferred to the 5′ end of the vRNA molecule by the guanylyltransferase activities of nsP1, resulting in the 7meGppA type 0 cap structure (11).

In response to the lack of preventatives or treatments, targeting the alphaviral replication machinery has been a popular approach for developing potential antiviral therapies. Capping of the genomic and subgenomic vRNAs is vital for successful viral replication, as mutations that completely inhibit capping of the viral RNA render the virus noninfectious. Thus, because nsP1 is responsible for the alphaviral capping process, it has been a popular target for antiviral research. In particular, a number of compounds have been developed that inhibit nsP1 capping activity and reduce viral replication *in vitro*, but, to date, none have been tested for efficacy against alphaviral infection *in vivo* (12–15). In addition to the development of drugs against nsP1 activity, multiple residues in nsP1 have also been identified as determinants for alphaviral virulence; however, the impact of these residues on alphaviral capping efficiency has never been delineated. The SINV nsP1/nsP2 cleavage mutant T538I has been shown to determine pathogenicity in mouse models of infection by altering nonstructural polyprotein processing and the virus’ sensitivity to interferon (16, 17). More recently, a group of six mutations in the nsP1 of RRV have also been shown to attenuate alphaviral disease in mice, although the mechanism of attenuation and the impacts of these mutations on alphaviral replication have yet to be fully characterized (18, 19). These studies illustrate the significance of nsP1 to alphaviral infection and pathogenicity but have yet to determine the importance of alphaviral capping efficiency and the production of the noncapped genomic RNAs (ncgRNAs) to *in vivo* infection.

While capping of the viral RNA is critical to viral protein expression and viral replication, we have previously shown that the genomic vRNAs are not universally capped and that a significant proportion of the alphaviral genomic RNA produced and packaged during infection lack the 5′ cap structure (20). In addition, our recently published study showed that the proportion of ncgRNAs produced during SINV infection could be altered using point mutations in nsP1 to modulate capping activity (21). Specifically, incorporating a D355A mutation in the nsP1 of SINV resulted in increased capping efficiency and, therefore, decreased ncgRNA production, relative to that of wild-type SINV. Alternatively, an N376A mutation in nsP1 resulted in decreased capping efficiency and increased ncgRNA production. By utilizing these mutations to alter ncgRNA production, we were able to show that increasing the capping efficiency of nsP1 was detrimental to SINV infection in tissue culture models of infection, while decreasing nsP1 capping efficiency did not significantly affect viral titer or overall replication.

However, the presence or lack of a phenotype *in vitro* is not always indicative of what will occur during infection *in vivo*. As such, the goal of this study was to determine the effect of altered ncgRNA production on alphaviral pathogenesis by using the previously described nsP1 capping mutants in a mouse model of infection. The data presented here show that modulating ncgRNA production through the use of the D355A and N376A point mutations to alter nsP1 capping efficiency in nsP1 has a profound impact on alphaviral pathogenesis. In particular, decreasing capping efficiency resulted in increased sensitivity to type I interferon (IFN) and a slight decrease in mortality. Surprisingly, increasing capping efficiency resulted in almost complete abrogation of morbidity and mortality, despite showing increased resistance to type I IFN, due to reduced immune infiltration and production of inflammatory cytokines in the brain. Collectively, our findings indicate that the ncgRNAs are important in determining the host immune response to viral infection and play a critical novel role in alphaviral pathogenesis.

## RESULTS

### Altering capping efficiency is detrimental to viral growth kinetics in neurovirulent SINV *in vitro*.

Given our previously reported findings describing the molecular phenotypes of the nsP1 mutants in tissue culture models of infection, we were interested in characterizing how altering ncgRNA production impacted SINV infection *in vivo*. However, our previous characterizations of the capping mutants were done using a Toto1101-derived strain of SINV, which is tissue culture adapted and does not cause disease in adult wild-type mice. Rather than rely on very young mice or mouse models with deficiencies in IFN competency to assess pathogenesis, we elected to change the SINV strain used to allow assessments in adult wild-type mice. Thus, we incorporated the D355A and N376A nsP1 mutations into the AR86 background of SINV, a neurovirulent strain capable of causing lethal disease in an adult mouse model.

**FIG 1:**
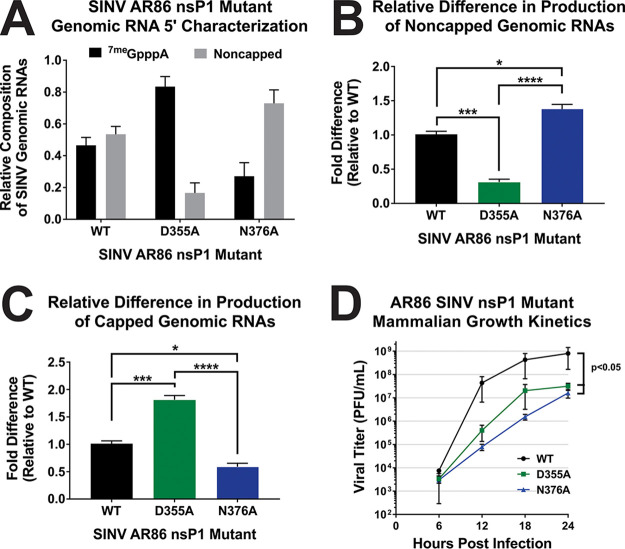
Point mutations in nsP1 of AR86 SINV result in changes in capping efficiency and negatively impact infection in mammalian cells. (A) Quantitative assessment of SINV RNAs produced during infection of BHK-21 cells with either wild-type (WT) SINV or either of the nsP1 mutants at an MOI of 5 PFU/cell. RNA was collected at 16 hpi and treated as described in Materials and Methods. Graphs depict the relative quantities of noncapped (B) or capped (C) genomic RNAs produced during infection of BHK-21 cells with wild-type SINV or either of the nsP1 mutants at 16 hpi. (D) One-step growth kinetics of the individual capping mutants and the parental wild-type SINV in BHK-21 cells infected at an MOI of 5 PFU/cell. All the quantitative data shown represent means of results from three independent biological replicates, with error bars representing standard deviations of the means. Statistical significance was determined by analysis of the area under the curve. *, *P* < 0.05; **, *P* < 0.01; ***, *P* < 0.001; ****, *P* < 0.0001.

To confirm that these point mutations had phenotypes in the AR86 background similar to the previously used Toto1101-derived background, the proportions of capped and noncapped genomic RNAs were quantified in BHK-21 cells at 16 h postinfection (hpi) (Fig. 1A to C). For wild-type AR86 SINV, approximately half of the genomic RNA was found to be capped. This is consistent with what was previously found for the Toto1101-derived SINV, which also exhibited a capped/noncapped ratio of 1:1 at the equivalent time during infection (20). Specifically, the D355A nsP1 mutation resulted in production of an RNA population consisting of 4-fold more capped RNA than noncapped RNA, whereas the N376A nsP1 mutation produced a population with 4-fold more noncapped RNA than capped RNA (Fig. 1A). Compared to wild-type AR86, the D355A mutant produced significantly more capped vRNA and significantly less ncgRNA (Fig. 1B and C). Conversely, the N376A mutant produced significantly less capped vRNA and significantly more ncgRNA than wild-type SINV. Although the absolute magnitude by which capping efficiency was affected by the nsP1 mutations appears to be different in the AR86 strain from that observed for the previously used Toto1101-derived strain, the trend of the D355A mutation increasing capping efficiency and the N376A mutation decreasing capping efficiency remained the same (21).

In addition to confirming the impact of the nsP1 mutations on vRNA capping in the AR86 background, viral growth kinetics were also assessed in BHK-21 cells (Fig. 1D). Similar to what we have reported previously, increasing the capping efficiency of nsP1 with the D355A mutation resulted in an approximately 1.5-log decrease in viral titer over the course of infection. Likewise, decreasing capping efficiency with the N376A mutation also resulted in a significant decrease in viral growth kinetics. While the phenotype associated with the N376A mutant was more dramatic in the AR86 strain than what was reported in our previous study, this might be explained by the fact that the AR86 strain of SINV is not adapted for replication in tissue culture unlike the previously used Toto1101 strain. Therefore, the impact of the N376A mutation on replication is likely exacerbated in tissue culture systems for the AR86 strain, leading to the significantly reduced viral growth kinetics seen in Fig. 1D.

**FIG 2:**
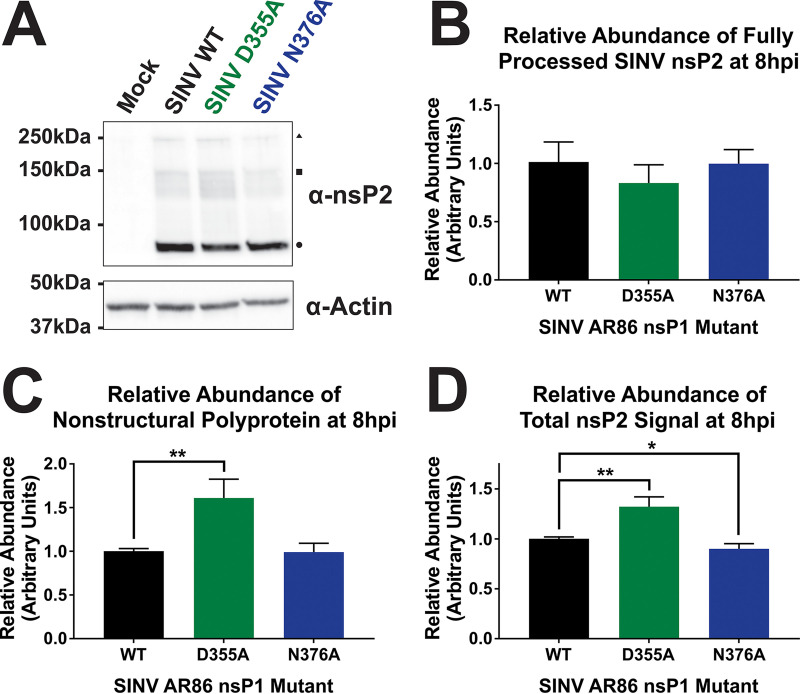
Increasing capping efficiency increases translation of SINV AR86 nonstructural polyprotein. (A) BHK-21 cells were infected with either wild-type SINV or one of the nsP1 capping mutants at an MOI of 5 PFU/cell. Abundance of nsP2 was then assessed at 8 hpi by Western blotting. ▴, Nonstructural polyprotein band. ■, p23 polyprotein intermediate band (as determined by molecular weight). ●, Fully processed nsP2 band. Actin is shown as the loading control. (B to D) Densitometric quantification of fully processed nsP2 protein (B), nonstructural polyprotein (C), and total nsP2 signal (D) normalized to actin levels at 8 hpi. All the quantitative data shown represent means of results from three independent biological replicates, with error bars representing standard deviations of the means. Statistical significance was determined using Student's *t* test.

To further confirm that the nsP1 mutants did not introduce gross life cycle defects in the AR86 background, viral translation was measured. To assess how the nsP1 capping mutations affected viral translation, BHK-21 cells were infected with either wild-type SINV or one of the nsP1 capping mutants, and the relative expression of the SINV nsP2 protein was detected via Western blotting (Fig. 2A). Densitometry analysis of the fully cleaved form of nsP2 revealed no quantitative differences across the SINV AR86 nsP1 mutant strains (Fig. 2B). Despite no apparent differences in fully processed nsP2 levels, increased levels of nonstructural polyprotein were detected in the SINV nsP1 D355A mutant via comparative densitometry (Fig. 2C). Furthermore, comparing the total signal detected by the anti-nsP2 polyclonal serum reveals slightly enhanced nonstructural gene expression in the D355A nsP1 mutant, consistent with our previous observations using the Toto1101-derived nsP1 mutants. These data are suggestive of increased or ongoing nonstructural protein synthesis in the D355A background. As supported by the equivalent levels of fully processed nsP2, the processing of the nonstructural polyprotein likely occurs at a rate that is unaffected by the level of translation of the polyprotein. Thus, increasing the capping efficiency of the nsP1 protein led to increased nonstructural gene expression. Unlike what was previously found, the N376A decreased capping mutant showed no difference in viral translation compared to wild-type SINV.

On the basis of our previous examinations of the Toto1101-derived SINV nsP1 mutants, we postulate that differences in viral translation are more pronounced during the earliest stages of infection. Previously we utilized nanoluciferase reporter viruses to quantitatively assess viral gene expression in a highly sensitive manner. Unfortunately, the inclusion of a nanoluciferase reporter into the nsP3 protein of SINV AR86 has been found to be highly attenuating, precluding the detailed quantitative assessment of early SINV AR86 translation.

**FIG 3:**
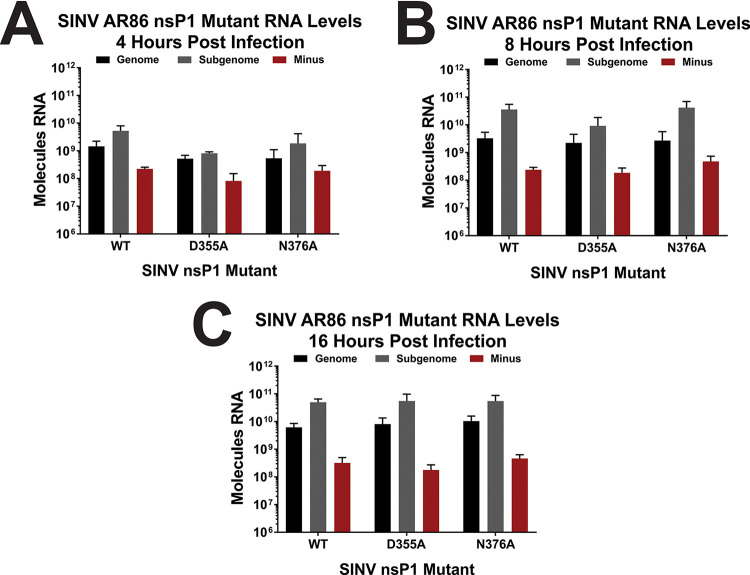
Altering capping efficiency does not impact AR86 SINV vRNA synthesis. BHK-21 cells were infected with either wild-type SINV or one of the nsP1 capping mutants at an MOI of 5 PFU/cell. Absolute quantities of the genomic, subgenomic, and minus-strand vRNAs produced at 4 (A), 8 (B), and 16 (C) hpi were determined by qRT-PCR. All the quantitative data shown represent means of results from three independent biological replicates, with error bars representing standard deviations of the means. Statistical significance was determined using Student's *t* test.

In addition to viral translation, viral RNA synthesis/accumulation was also assessed to determine if the nsP1 capping mutations affected RNA kinetics in the AR86 strain of SINV. Similar to what was shown in the Toto1101-derived background, there were no significant differences for any of the vRNA species produced by either nsP1 mutant at any of the measured time points compared to wild-type SINV (Fig. 3A to C) (21).

Taken together, we can conclude that the D355A and N376A nsP1 mutations affect capping efficiency and viral nonstructural protein expression in the AR86 strain of SINV in a manner that is similar to that of the previously used Toto1101-derived background. We can also conclude that the decreases seen in viral titer between the nsP1 capping mutants and wild-type SINV are not due to deficits in viral translation or RNA synthesis but rather are due to changes in the proportion of ncgRNA produced by each mutant. Overall, the recapitulation of the original D355A and N376A capping phenotypes in the AR86 background provided a means by which the biological impact of the ncgRNAs on viral infection and pathogenesis could be assessed using an adult wild-type mouse model. However, since our data suggest that the early events of the viral life cycle are altered by modulating capping efficiency, we decided to first characterize the engagement of the AR86-derived nsP1 mutants with the host innate immune response at the cellular level prior to utilizing a small-animal model of SINV infection (21).

### Modulating ncgRNA production alters the host type I interferon response to SINV infection.

**FIG 4:**
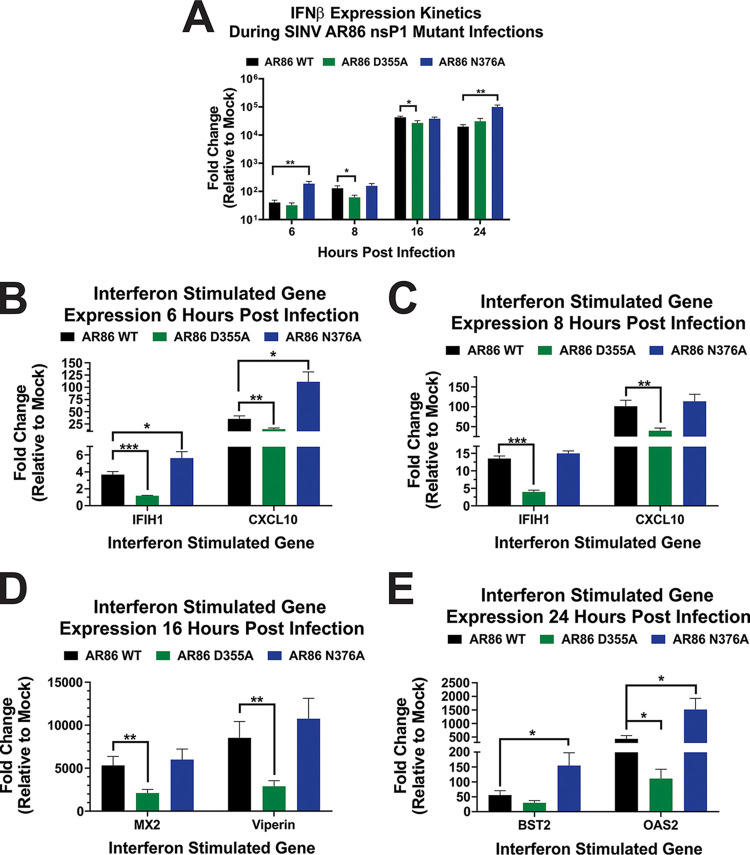
Production of type I interferon and ISGs in response to SINV nsP1 capping mutants. L929 cells were infected at an MOI of 10 PFU/cell with either wild-type SINV or an individual capping mutant. Cell lysates were collected at 6, 8, 16, and 24 hpi, and transcript expression levels for IFN-β (A) and the selected ISGs (B to E) were determined by qRT-PCR for their respective times postinfection. Data were normalized to GAPDH and nsP1 and calculated relative to uninfected controls. All the quantitative data shown represent means of results from three independent biological replicates, with error bars representing standard deviations of the means. Statistical significance was determined by Student's *t* test.

The capacity of the host cell to detect viral infection and mount an antiviral response by upregulating interferon and interferon-stimulated gene (ISG) expression is an important aspect of viral infection. As such, we were interested in determining whether changes in viral capping efficiency would impact the stimulation of the host type I IFN response. To assess the extent to which the nsP1 mutants elicited an interferon response, interferon-competent L929 cells were infected with either wild-type SINV or one of the capping mutants, and then the expression of IFN-β was measured at the transcriptional level using quantitative reverse transcription-PCR (qRT-PCR) (Fig. 4A). For this experiment, in addition to quantitatively assessing IFN-β transcripts throughout the viral life cycle, we also quantified the transcript abundance of select ISGs with well-established times of maximal expression after viral infection so that we could more accurately determine the host antiviral response over the course of infection (22). As such, CXCL10 and IFIH1 were used to determine the ISG expression at 6 and 8 hpi, Viperin and MX2 at 16 hpi, and OAS2 and BST-2 at 24 hpi (Fig. 4B to E).

Infection with the D355A mutant resulted in significantly reduced IFN-β expression compared to that of wild-type SINV at 8 and 16 hpi, while N376A infection resulted in significantly greater IFN-β expression at 6 and 24 hpi (Fig. 4A). Regarding ISG transcript levels, infection with the N376A mutant resulted in significantly increased ISG expression compared to that of wild-type SINV at 6 hpi, but transcript abundance was roughly equivalent to that of wild-type infection at 8 hpi (Fig. 4B to D). However, by 24 hpi, ISG expression levels once again were significantly increased (Fig. 4E). In contrast, ISG expression levels in response to the D355A mutant was significantly decreased compared to that of wild-type SINV until 24 hpi, where OAS2 expression was still significantly decreased, but BST2 expression was similar to that of the wild type.

Overall, infection with the increased capping mutant, D355A, led to a mostly decreased host antiviral response compared to what was seen during wild-type infection, while the decreased capping mutant, N376A, elicited a response that was mostly increased compared to that of wild-type SINV. Taken together, these results illustrate that modulating ncgRNA production has a significant impact on the induction of the host type I IFN response.

### Sensitivity to type I IFN correlates with capping efficiency in tissue culture.

**FIG 5:**
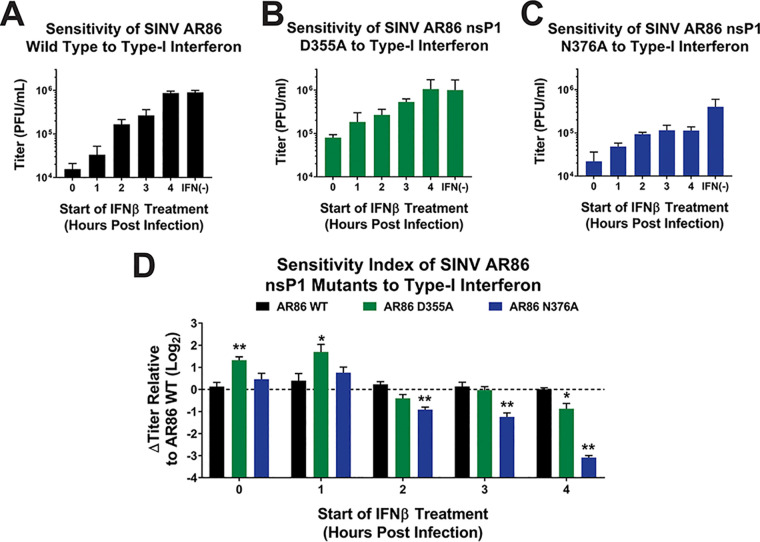
Analysis of SINV sensitivity to type I interferon. L929 cells were infected with either wild-type SINV or an individual capping mutant at an MOI of 10 PFU/cell. At the indicated times postinfection, 20 IU of recombinant type I IFN was added to the growth medium, and the cells were incubated for a period of 24 h. (A to C) Viral titers were quantified via plaque assay. (D) The relative sensitivity of the viruses was determined by comparing their growth to that of untreated controls. All the quantitative data shown represent means of results from three independent biological replicates, with error bars representing standard deviations of the means. Statistical significance was determined by Student's *t* test. *, *P* < 0.05. **, *P* < 0.01.

Given that altering capping efficiency affected viral gene expression and altered the induction of the host type I IFN response, we were interested to see if changes in ncgRNA production affected the virus’ sensitivity to IFN treatment. In other words, we have shown that altering capping efficiency impacts how much IFN is produced, but does altering SINV capping efficiency affect the virus’ capacity to resist IFN treatment when the cells are treated with equal amounts of type I IFN? To determine whether changes in ncgRNA production affected the sensitivity of the nsP1 mutant viruses to exogenous type I IFN, IFN-competent L929 cells were infected with either the wild type or a SINV nsP1 mutant virus, recombinant type I IFN was added at 0, 1, 2, 3, or 4 h postinfection, and viral titer was measured at 24 hpi (Fig. 5A to C) (23).

During wild-type SINV infection, viral titer was reduced by ∼2 logs when IFN was added at 0 hpi, with maximum titer increasing regularly as the time of IFN addition was delayed. IFN treatment at 4 hpi did not impact SINV AR86 replication, as viral titers were equivalent to those of the no IFN control (Fig. 5A). The D355A mutant, which has increased capping efficiency and viral gene expression, was found to be significantly more resistant to type I IFN treatment early during infection than wild-type SINV, with the viral titer being reduced by only ∼1 log when IFN was added at 0 hpi (Fig. 5B). As with wild-type SINV, the maximal titer of the D355A mutant steadily increased as the addition of type I IFN was delayed to later times postinfection; however, the relative differences in titer between the IFN-treated infections and nontreated control indicate that the D355A mutant was significantly more resistant to the impacts of type I IFN overall. In contrast, the N376A mutant, which has decreased capping efficiency, was found to have wild-type-equivalent sensitivity to IFN when added at 0 and 1 hpi, but the N376A mutant remained sensitive to the addition of type I IFN at later times during infection (Fig. 5C). Surprisingly, even when IFN was added as late as 4 hpi, the titers of the N376A mutant remained decreased compared to those of the IFN negative control, revealing that the N376A mutant remains sensitive to IFN for a longer period of time than wild-type SINV.

To enable the comparative analysis of the impact of type I IFN on the viral infections of the capping mutants compared to wild-type infection, the differences in viral titers between the IFN-treated and the control infections for each virus and time point were calculated. The differences in titer found for each nsP1 mutant were then made relative to the corresponding differences observed for the wild-type infection (Fig. 5D). These data illustrate that during the early stages of viral infection, the D355A nsP1 mutant is approximately 2-fold more resistant to type I IFN than wild-type SINV AR86; however, by 2 hpi the advantage had largely waned, and the level of IFN resistance was similar to that observed during wild-type infection. Similarly, comparing the resistance of the N376A mutant to wild-type SINV AR86 further reveals that decreasing capping efficiency correlates with significantly increased sensitivity to type I IFN up to at least 4 h postinfection.

It is interesting that, in the absence of IFN treatment, infections of the IFN-competent L929 cells with wild-type SINV or the D355A mutant resulted in roughly equivalent viral titers at 24 hpi. This is different from what was observed previously in BHK-21 cells, where the D355A mutation resulted in significantly decreased viral titer (Fig. 1D). This difference in phenotypes between the two cell lines is likely due to the fact that the L929 cells are IFN competent and will produce IFN in response to viral infection while BHK-21 cells are incapable of doing so, resulting in differential viral replication rates due to the host response to viral infection in conjunction with the apparent differences in IFN sensitivity.

Overall, the sensitivity of the SINV nsP1 mutants to IFN reflects the differences seen in capping efficiency. Increased capping efficiency resulted in the D355A mutant being more resistant to type I IFN early during infection compared to wild-type SINV. Likewise, decreased capping efficiency resulted in the N376A mutant being more sensitive to type I IFN. This indicates that both the viral response to type I IFN and the virus’ ability to mitigate the effects of IFN expression on viral replication are altered depending on the level of ncgRNA produced during infection.

### Increasing capping efficiency significantly attenuates neurotropic SINV in a mouse model.

**FIG 6:**
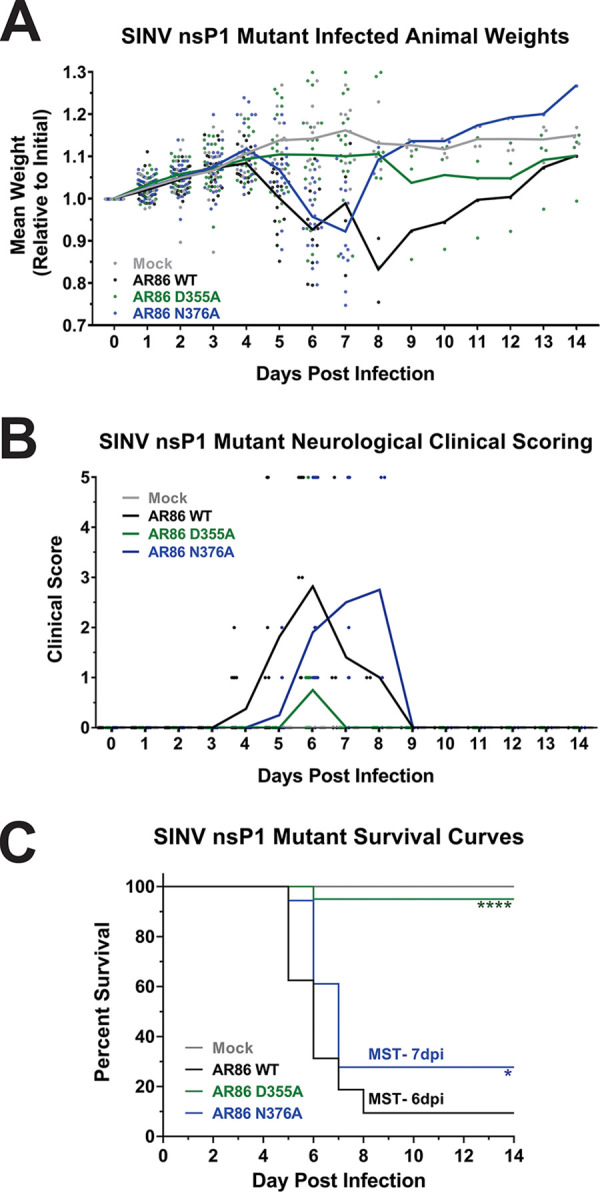
Increased vRNA capping efficiency reduces SINV AR86 mortality and pathogenesis. Four-week-old male and female C57BL/6J mice were either mock infected or infected with 1,000 PFU of SINV AR86 wild type, D355A, or N376A via rear footpad subcutaneous inoculation. Each data point represents a single animal from either experimental replicate. The experimentally infected mice were assessed over a 14-day period. (A) Animals were weighed twice daily. Weights are shown relative to initial weight after being infected. (B) Mice were scored based on a 1 to 5 scale for neurological response. (C) Kaplan-Meier analysis indicates the WT median survival time (MST) at ∼6.4 days and the N376A mutant MST at ∼7 days. The *P* values indicated were determined by the log rank test. *, *P* < 0.05; ****, *P* < 0.0001. Data shown were pooled from 2 independent experiments.

As the capacity to avoid the elicitation of the host innate immune response and the capacity to disregard the effects of the host type I IFN response are vital to alphaviral replication and pathogenesis, the above-described data suggested that altering ncgRNA production has profound effects on viral replication and pathogenesis *in vivo*. We hypothesized that, due to the nsP1 D355A mutant’s increased resistance to type I IFN and generally reduced activation of ISG expression, mice infected with the nsP1 D355A mutant would experience disease severity similar to that of wild-type SINV infection, perhaps with the mean survival time being decreased due to increased IFN resistance. Conversely, we hypothesized that mice infected with the N376A mutant would experience more mild disease and decreased mortality because of the mutant’s increased sensitivity to IFN and the greater expression of ISGs in response to infection in tissue culture models of infection. To test our hypothesis, we infected 4-week-old male and female C57BL/6 mice with 1,000 PFU of SINV AR86 wild type, nsP1 D355A, or nsP1 N376A via rear footpad subcutaneous inoculation. Mock-infected mice were inoculated with phosphate-buffered saline (PBS) in the same manner. When infected with wild-type SINV, adult C57BL/6 mice displayed significant weight loss as well as severe neurological symptoms, including rapid-onset paralysis of the limbs, blindness, and seizures at approximately day 6 postinfection (Fig. 6A and B). Infection with wild-type SINV also led to significant mortality, with infected mice having a mean survival time of ∼6 days postinfection (Fig. 6C). Likewise, mice experimentally infected with the decreased capping mutant N376A exhibited weight loss and neurological symptoms similar to those of wild-type-infected mice. However, the onset of disease in the N376A-infected mice was delayed compared to that of wild-type SINV, with neurological symptoms starting at 5 days postinfection (dpi) and the mean survival time being ∼7 dpi, a full day later than what was seen with wild-type SINV. In addition, a slightly greater proportion of mice survived when infected with the N376A mutant as opposed to wild-type SINV. This increase in survival may be due to the delay in the N376A mutant causing neurological symptoms, allowing the mice to be slightly older and, therefore, better able to resist severe, lethal encephalitis (24–26).

Surprisingly, the increased capping mutant virus D355A was significantly attenuated in mice. Compared to the previous two viruses, mice infected with the D355A mutant experienced minimal weight loss, milder neurological symptoms, and significantly reduced mortality, with all but one mouse surviving to the end of the study. Given that these mice did in fact show mild neurological and nonneurological symptoms and reduced weight gain compared to mock-infected mice, we concluded that the nsP1 D355A increased capping mutant virus is indeed capable of causing pathogenesis, although the severity of disease is significantly reduced compared to that of wild-type infection.

**FIG 7:**
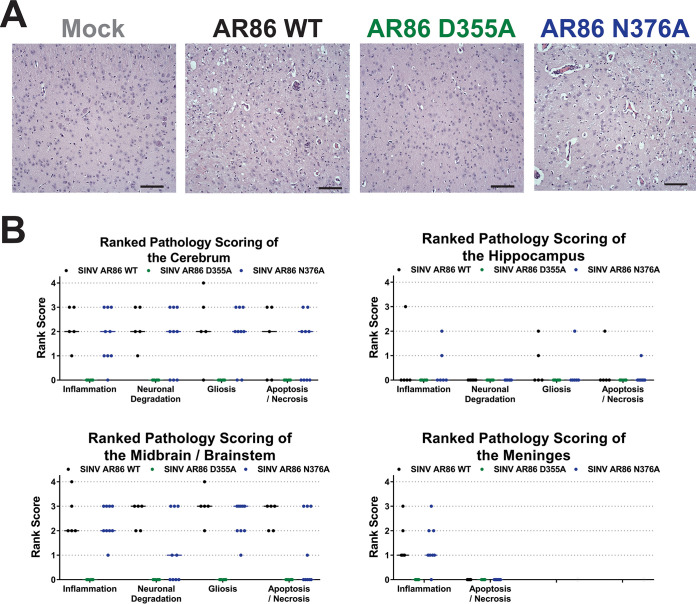
Increased capping efficiency leads to decreased pathology in the brain. (A) Representative H&E-stained sagittal sections of the midbrain (×20 magnification) from mock-, wild-type-, or capping mutant-infected mice at 7 dpi or at the time at which endpoint criteria were met. The brains of SINV wild-type- and N376A-infected mice show large amounts of perivascular cuffing, immune infiltration, and cell death not present in the mock- and D355A-infected mice. Scale bar, 0.1 mm. (B) Ranked pathology scoring of indicated sections of the brain from infected mice. Data points indicate scoring for each experimental animal, representing at least 5 biological replicates.

The trends seen in morbidity and mortality between the mice infected with wild-type SINV versus the capping mutants were further reflected in hematoxylin and eosin (H&E)-stained sections of the brains of infected and uninfected mice (Fig. 7). The brain sections of both wild-type and N376A-infected mice displayed numerous lesions consisting of lymphocytic meningitis; perivascular cuffing, which is indicative of immune cell infiltration; and neuronal apoptosis, which left open pockets in the tissue (Fig. 7A). In addition, mice infected with either wild-type SINV or the N376A mutant had significant pathology in terms of inflammation, neuronal degradation, and glial cell proliferation in multiple areas of the brain. Pathology was highest in the cerebrum and the midbrain/brainstem, but lesions were also found in the hippocampus and medulla oblongata of some mice (Fig. 7B). Conversely, the brains of the D355A-infected mice resembled those of mock-infected mice, with no immune infiltration, cell death, or other signs of pathology in any area of the brain. Given that viral killing of neurons is the speculated cause of encephalitis and paralysis in SINV infection, the differences in tissue damage and neuron death seen between the D355A mutant and the other two viruses were not unexpected, as the D355A-infected mice did not exhibit signs of encephalitis or limb paralysis (Fig. 6B) (27, 28).

Collectively, these data suggest that modulating the production of ncgRNA has significant impacts on alphaviral pathogenesis. Overall, the nsP1 N376A point mutation, which increased ncgRNA production through decreased capping efficiency, resulted in delayed disease progression and mortality compared to wild-type SINV *in vivo*. However, the severity of neurological symptoms and pathology in the brain were unaffected. In contrast, the nsP1 D355A point mutation, which decreased ncgRNA production through increased capping efficiency, resulted in significantly decreased mortality, mild neurological symptoms, and little to no pathology in the brain. While the presence of mild symptoms and a lack of weight gain in the D355A-infected mice do suggest that the virus was capable of trafficking to the brain and replicating, these data do not eliminate these as being possible reasons for the decreases in pathogenesis seen thus far.

### Attenuation of viral pathogenesis is not due to deficits in viral dissemination or replication.

Given that the D355A and N376A nsP1 mutants showed decreased viral titers in tissue culture model systems compared to wild-type SINV (Fig. 1D), we hypothesized that the reduction in mortality seen in Fig. 6 was due to poor viral replication, dissemination, or a change in virus tropism for the brain. To determine the impact of modulating capping efficiency on viral replication and to confirm that the nsP1 D355A mutant did in fact make it to the brains of infected mice, we measured viral titers at the site of inoculation and in the serum at 1 dpi as well as in the brain at 7 dpi. By comparing the viral titers of the nsP1 D355A mutant to those of wild-type SINV in these tissues, we were able to determine if decreasing ncgRNA production impacts viral pathogenesis by altering viral replication, dissemination, or tropism to the brain. If the D355A mutant had defective dissemination or tropism to the brain, then we would expect to see wild-type titers at the site of inoculation, and potentially in serum, but an absence of viral titer in the brain. Alternatively, if viral titers for the D355A mutant are significantly decreased in the ankle, serum, and brain, then this would suggest that the decreases seen in pathogenesis were due to poor viral replication and dissemination.

**FIG 8:**
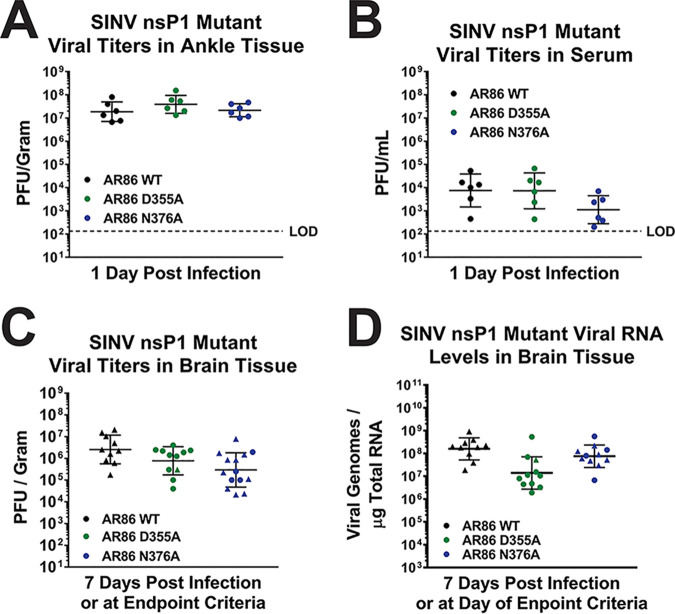
Viral replication is largely unaffected by altered capping *in vivo***.** (A to C) Tissues were harvested at the indicated times postinfection, and viral titer was determined via plaque assay. (D) Viral genomes were measured by qRT-PCR. The data points indicate the individual titers for each experimental animal, and the mean values shown are the geometric means of at least four biological replicates from two independent experiments, with the error bars representing the geometric standard deviations of the means. ▲, Mice that met endpoint criteria prior to day 7. Statistical significance was determined using Student's *t* test.

Surprisingly, in contrast to what was expected given our tissue culture data, viral titers in the ankle, serum, and brain were more or less equivalent between wild-type SINV and the two nsP1 mutants (Fig. 8). The similar titers found in the ankle between the nsP1 mutants and wild-type SINV show that the reduced pathogenicity of the nsP1 D355A mutant is not due to a defect in viral replication at the site of inoculation (Fig. 8A). Likewise, since the nsP1 D355A mutant had titers equivalent to wild-type levels in the serum, we can also conclude that viral dissemination was not negatively impacted (Fig. 8B). Finally, while the viral titers of the D355A and N376A mutant were both slightly decreased in the brain compared to wild-type SINV, the lack of a significant difference indicates that increasing capping efficiency did not alter viral tropism to the central nervous system (CNS) and that there is no overt defect in viral replication (Fig. 8C). Interestingly, while these results are different from what was previously observed during infection of BHK-21 cells (Fig. 1D), the trends seen in the serum and brain, where the N376A mutant has slightly decreased viral titers compared to those of wild-type SINV and the D355A mutant, were similar to what was seen with the L929 cells (Fig. 5).

To further complement our observations regarding the induction of the host innate immune response to infection (as in Fig. 4A), we quantitatively assessed IFN-β transcript levels in the ankles of the experimentally infected mice at 24 hpi. Despite differences in the overall magnitudes of type I IFN induction, we found no differences between our *in vitro* and *in vivo* assessments (see Fig. S1 in the supplemental material).

10.1128/mBio.02675-20.1 FIG S1 Quantitative analysis of IFN-β transcript levels in SINV AR86-infected ankle tissue. Ankle tissues harvested from either mock-infected or wild-type SINV AR86- or SINV AR86 nsP1 mutant-infected animals were harvested at 1 day postinfection and homogenized. Total cellular RNA was isolated from the tissue homogenates and assessed via qRT-PCR to determine the relative transcript abundances of the IFN-β gene. Quantitative data shown are the means from at least three biological replicates, with the error bars representing the standard deviations of the means. Statistical analysis of the data, by Student’s *t* test, indicated no significant differences between the SINV AR86-infected tissues regarding IFN-β transcript levels. Download FIG S1, TIF file, 0.5 MB. Copyright © 2020 LaPointe et al. 2020 LaPointe et al. This content is distributed under the terms of the Creative Commons Attribution 4.0 International license.

The dissemination of the SINV mutants to the brain was further confirmed when viral RNA levels in the brain were measured (Fig. 8D). While the nsP1 D355A mutant did exhibit slightly lower vRNA abundance in the brain compared to that in wild-type SINV, it was not found to be a statistically significant difference and is likely an artifact due to differences in when the brain tissue was collected. While the majority of the D355A-infected mice survived to 7 dpi when the brain tissue was collected, all of the wild-type-infected mice met endpoint criteria prior to 7 dpi. As such, the adaptive immune response in the D355A-infected mice that survived to 7 dpi may have started to clear some of the infected cells serving as viral RNA reservoirs from the brain, resulting in the decreased vRNA abundance compared to that of the wild-type-infected mice that did not survive long enough to mount a similar response. N376A RNA levels in the brain were also found to be equivalent to that of wild-type SINV. These results indicate that while the D355A and N376A nsP1 point mutations were capable of reducing viral titer in tissue culture, they were not detrimental to viral replication in mouse models of infection.

Taken together, these data suggest that both the increased capping virus D355A and the decreased capping virus N376A were capable of trafficking to the brain from the site of inoculation and were capable of replicating to high titers within the brains of experimentally infected adult mice. It is also interesting that neither viral titer nor viral genomic RNA abundance correlated with death, as there were multiple mice that survived infection that had greater viral titer and vRNA abundance in the brain than mice that died. Overall, these results led us to conclude that the attenuation of pathogenesis seen in the D355A mutant was not due to deficits in either viral replication or tropism.

### Increasing capping efficiency does not affect viral induction of neuronal apoptosis.

**FIG 9:**
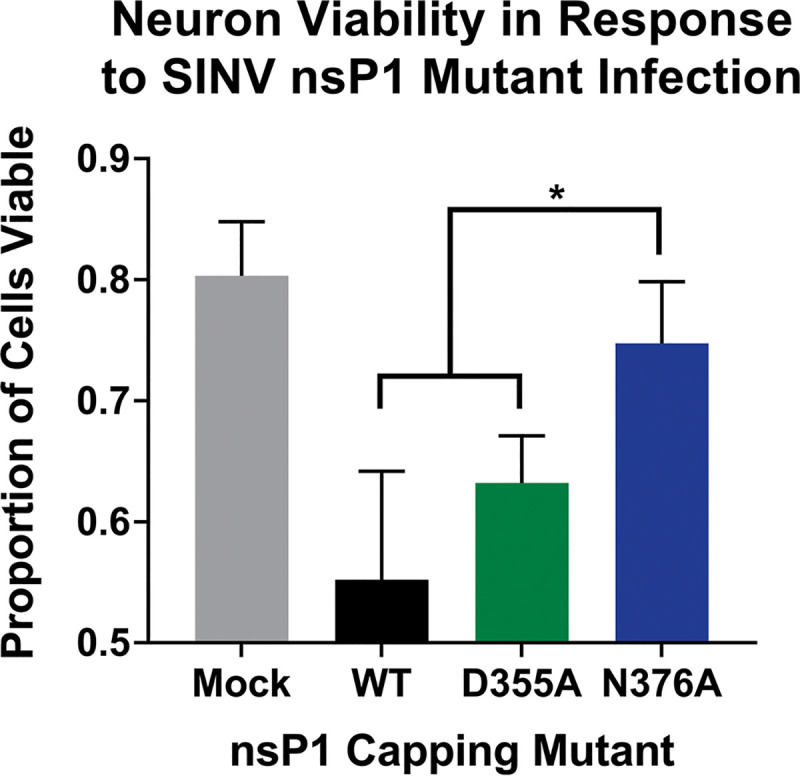
Neuron viability increased with decreased capping efficiency. SK-N-BE(2) neurons were infected at an MOI of 30 PFU/cell with either wild-type SINV or an individual capping mutant. Cell viability was determined at 24 hpi using ethidium bromide/acridine orange staining and is represented as the proportion of viable cells out of total cells counted. A minimum of 100 total cells per well were counted using ImageJ. All the quantitative data shown represent means of results from three independent biological replicates, with error bars representing standard deviations of the means. Statistical significance was determined by Student's *t* test.

Since we did not find any significant differences in viral replication or tissue tropism/dissemination, we next determined if decreased mortality in the D355A mutant was due to an altered capacity to induce neuronal death, as virally induced apoptosis of neurons in the brain, brainstem, and spinal cord have been shown to be responsible for the severe neurological symptoms that arise during SINV infection (27–30). In addition, our previous study characterizing the nsP1 capping mutants in tissue culture showed that both the D355A and N376A mutant demonstrated increased cell viability in BHK cells compared to wild-type SINV, supporting the possibility that the D355A mutant has differences in cell viability in neurons as well (21). In light of our previous study as well as the striking difference in cell death between the D355A- and wild-type-infected brains (Fig. 7), we hypothesized that increasing capping efficiency would decrease the virus’ capacity to kill neurons. To test this hypothesis, we infected SK-N-BE(2) cells with either wild-type SINV or one of the capping mutants and determined cell viability 24 h after infection using ethidium bromide and acridine orange staining. Infection with the decreased capping N376A mutant resulted in significantly greater cell viability than that of either wild-type SINV or the D355A mutant, which both exhibited roughly similar levels of cell death (Fig. 9). Interestingly, these results suggest that increasing ncgRNA production leads to increased cell survival in tissue culture models of infection, while decreasing ncgRNA production does not seem to impact the virus’ capacity to kill neurons in tissue culture models of infection. Given this, we can conclude that the differences seen in tissue damage between the D355A and wild-type SINV infections in Fig. 7 are not solely due to deficits in the D355A mutant’s capacity to kill neurons. Instead, these results suggest that the neuronal death seen during SINV infection in mice is largely due to the host antiviral inflammatory response rather than direct cell death due to infection.

### Differential ncgRNA expression alters the immune response to infection.

**FIG 10:**
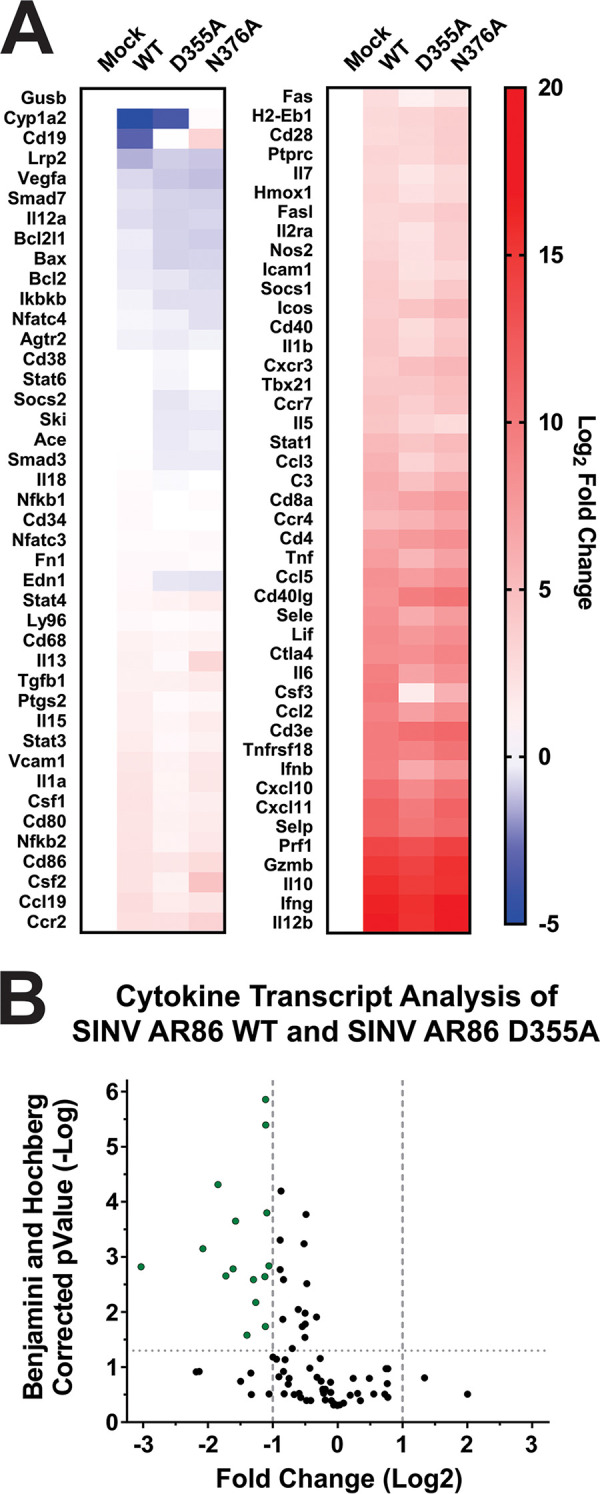

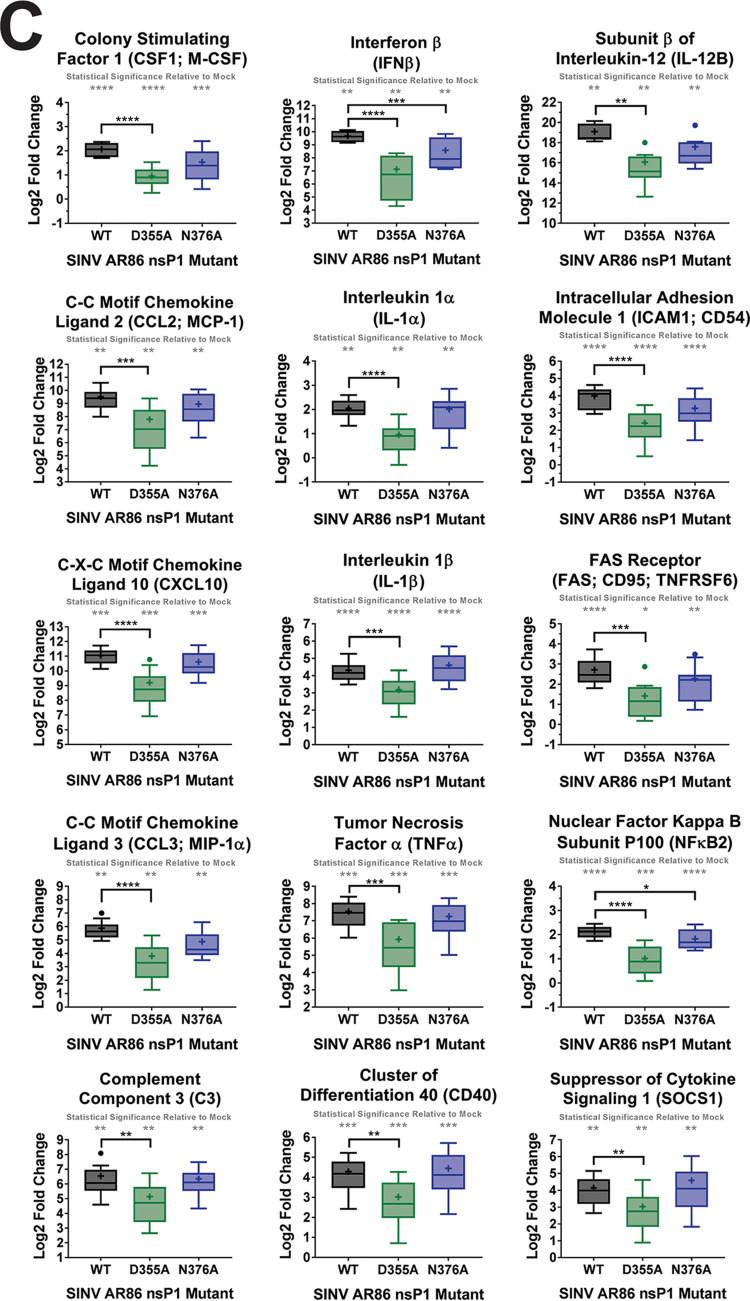
Increased viral capping efficiency results in reduced expression of proinflammatory genes in the brain. (A) Cytokine transcript levels in the brain at 7 dpi were measured by qRT-PCR. Data were normalized to GAPDH and calculated relative to uninfected controls. (B) Volcano plot showing the fold change in transcript expression between wild-type SINV and the D355A mutant. Green points are transcripts that have greater than a 2-fold change in expression and are significant according to the Benjamini and Hochberg-corrected *P* value. (C) Cytokines and chemokines whose expression was significantly increased compared to that of uninfected controls and exhibited a significant difference in expression between wild-type SINV and the D355A mutant was greater than 2-fold. All the quantitative data shown represent means of results from at least three independent biological replicates, with center lines representing the median, plus signs representing the mean, boxes representing the interquartile range, error bars representing standard deviation of the means, and filled circles representing outliers, as determined by Tukey’s method. Statistical significance was determined by Student's *t* test.

Because the differences in morbidity and mortality between the D355A nsP1 mutant- and wild-type SINV-infected mice could not be explained by reductions of viral titer or the capacity to induce neuronal death, we next questioned whether differences in pathogenesis could be due to an altered host immune response. Given the reduced immune infiltration and inflammation seen in the D355A-infected mice compared to the wild-type- or N376A-infected mice (Fig. 7), we expected infection with the D355A mutant to also result in the decreased expression of proinflammatory genes. To survey the immune response to SINV nsP1 mutant virus infections, RNA was isolated from whole-brain homogenates of infected mice at 7 dpi or upon meeting endpoint criteria, and the levels of select cytokine and chemokine transcripts were measured via a qRT-PCR-based array. Out of the transcripts measured, 50 were found to be significantly increased in wild-type-infected versus mock-infected mice (Fig. 10A). When wild-type and D355A infections were compared, we found 15 inflammatory cytokines and chemokines whose expression was determined to be significantly decreased (using a Benjamini-Hochberg corrected *P* value) by a magnitude greater than 2-fold (Fig. 10B). Individual box and whisker plots of these statistically significant transcripts can be found in Fig. 10C. These included chemokines involved in recruiting innate immune cells, such as CCL2, CCL3, and CXCL10, as well as important drivers of inflammation, like IFN-β, interleukin-1β (IL-1β), and tumor necrosis factor alpha (TNF-α). In particular, the expression of CCL2 and CCL3 has been highly correlated with areas of the brain experiencing high levels of gliosis and apoptosis, which is consistent with the differences in pathology scoring in those areas between the D355A mutant and wild-type SINV (Fig. 7B) (31). Interestingly, several of the proteins that had decreased expression in the D355A-infected mice compared to wild-type-infected mice were identified by gene ontology as being part of the extrinsic apoptotic pathway (32, 33). The identification of proteins involved in apoptosis, specifically Fas, IL-1α, IL-1β, and TNF-α, is consistent with the significant decrease in neuronal apoptosis seen in the H&E-stained sections of mice infected with the D355A mutant. Surprisingly, IFN-γ expression, which was previously shown to be important for noncytolytic clearance of virus from neuronal cells, was not found to be significantly different in either mutant compared to that of the wild type (Fig. 10A) (34). As expected, the decreases seen in the expression of these chemokines and proinflammatory cytokines are consistent with the reduced immune infiltration and tissue damage seen in Fig. 7.

Overall, these data show that the D355A increased capping mutant has significantly reduced pathogenicity in a wild-type mouse model of infection, correlating with decreased expression of multiple proinflammatory molecules at the transcriptional level. Furthermore, the above-described data suggest that it is the host response rather than viral replication *per se* that determines the extent of alphaviral pathogenesis. This is supported by the finding that the wild-type and D355A viruses both had roughly equivalent viral titers in the brain, yet wild-type SINV had significantly increased proinflammatory cytokine transcript expression compared to that of the D355A mutant. While transcript levels are not synonymous with protein levels, the correlation between the expression of antiviral transcripts and levels of inflammation in the brain suggest that antiviral protein expression follows the same trends. Taken together, it can be concluded that decreased ncgRNA production via increased capping efficiency leads to an altered host immune response, which in turn shapes alphaviral pathogenicity.

## DISCUSSION

### Altering capping efficiency changes viral sensitivity to and activation of host type I IFN.

For Sindbis virus, the sensitivity of the virus to type I IFN is largely dependent on the translation of the nonstructural proteins, which are responsible for interfering with the IFN signaling pathway and for shutting down host transcription (35–38). When capping efficiency was increased with the D355A mutant, nonstructural gene expression was also found to be increased, although the magnitude was not as drastic as that previously seen with the Toto1101-derived background (Fig. 2). However, this may not necessarily be representative of what happens early during viral infection, where the magnitude of effect is likely to be more pronounced. While we were previously able to characterize early viral translation in the Toto1101-derived background using a nanoluciferase reporter incorporated into nsP3, the construction of the identical reporter in the AR86 background resulted in significant attenuation. However, given that we have previously shown that the differences in viral translation for the D355A mutant were more pronounced early during infection and that increasing capping was correlated with increased genomic translation in both viral backgrounds (Fig. 2C), it is likely that the D355A mutant in the AR86 background would also follow this trend (21). Increased translation of the viral proteins would allow the virus to more quickly shut down IFN signaling pathways, host PAMP sensors, and host transcription and translation, which would explain why the D355A mutant showed reduced IFN-β and ISG expression and greater resistance to IFN-β treatment (23, 39). In addition, the altered IFN-β and ISG expression seen with the capping mutant likely reflects changes in the virus’ abilities to both avoid detection by the host and suppress the cellular antiviral response (36). Because IFN-β and ISG transcript abundance was normalized to viral RNA levels, we know that the differences seen in antiviral transcript expression are not simply due to differences in viral replication or the amount of vRNA present. Rather, the changes observed in IFN-β and ISG expression for the D355A and N376A nsP1 mutants are likely due to both differences in PAMP production and their abilities to shut off host transcription and translation. This is supported by the correlation between the production of ncgRNAs, which are viral PAMPs, and IFN-β/ISG expression (Fig. 1B and 4, 40, 41). The fact that the D355A mutant showed increased resistance to type I IFN even when IFN was added concurrently with the virus also demonstrates how increasing capping efficiency allows the virus to more readily mitigate the effects of the IFN response. The D355A mutant’s increased resistance to IFN at such an early time point suggests that, in addition to increased translation, the mutation also affects how quickly the virus progresses through the viral life cycle. Increasing the production of capped genomic vRNA could give the virus a head start on translation of the viral proteins responsible for shutting down the host IFN response, allowing the D355A mutant to be more resistant to IFN treatment early during infection.

### Noncapped genomic vRNAs are critical for SINV pathogenesis in mice.

The ability to resist and shut down the type I IFN response has been shown to be one of the major determinants of virulence for SINV. For example, the AR86 strain of SINV is a virulent strain known to efficiently suppress the type I IFN response and cause lethal neurotropic disease in adult mice. The genetically similar Girdwood strain only partially inhibits the type I IFN response and, thus, is avirulent in adult mice (17). Given the finding that the D355A mutant was both more resistant to type I IFN treatment and resulted in decreased ISG production compared to the wild-type virus, we were surprised to find that this mutant did not cause severe disease or mortality in mice. Equally surprising was the result that infection with the N376A mutant was similarly as severe and lethal as infection with wild-type SINV, despite N376A being more sensitive to IFN treatment and stimulating more ISG expression *in vitro*. Taken together, these findings suggest that there is a balance between inhibiting and activating the IFN response, which results in pathogenesis, and that tipping the scales too far in either direction causes the virus to become avirulent. Furthermore, our results indicate that ncgRNAs play a critical role in determining whether the virus is neurovirulent or avirulent, as decreasing ncgRNA production with the D355A mutation resulted in significant decreases in morbidity and mortality, while increasing ncgRNA production with the N376A mutation resulted in fully neurovirulent virus and lethal disease.

Although the D355A mutation had a much more striking impact on morbidity and mortality, the N376A mutation also had noticeable impacts on alphaviral pathogenesis, namely, a delay in the onset of symptoms, an increased mean survival time, and a moderate increase in overall survival compared to that of wild-type SINV. The idea that inhibiting capping efficiency is detrimental to alphaviral infection is not novel, and multiple compounds and drugs have been developed to specifically target nsP1 capping activity (12–15). Our results do not negate the idea that decreasing or inhibiting capping efficiency is an effective means of combating alphaviral infection but rather suggest that there is a threshold that needs to be reached before decreasing capping efficiency will be significantly detrimental to viral pathogenesis. Likewise, our mortality studies imply that the virus is much more sensitive to increasing capping efficiency and that this novel approach is more effective in limiting the severity of viral disease.

During SINV infection, the development of severe encephalitis, which leads to paralysis and death, is caused by the extensive apoptosis and necrosis of neurons in the brain and CNS, which is believed to be virally induced (27, 30, 42, 43). While apoptosis is the fate of many infected cells and neurons, a significant portion of uninfected neurons are killed during viral infection due to glutamate excitotoxicity (44). The lack of apoptosis and necrosis seen in the brains of mice infected with the D355A mutant (Fig. 7) suggests that, during *in vivo* infection, decreasing ncgRNA production by increasing nsP1 capping efficiency leads to decreased viral induction of neuronal apoptosis and, therefore, decreased glutamate release, resulting in the reduced death of infected and uninfected neurons. However, when tested in tissue culture cells, infection with the nsP1 D355A mutant resulted in wild-type levels of neuronal death, while infection with the N376A mutant, which showed extensive signs of neuronal apoptosis and necrosis *in vivo*, resulted in significantly increased neuron survival. Since increasing capping efficiency did not seem to impact the virus’ ability to induce neuron death in tissue culture, these data suggest that the apoptosis and necrosis of neurons seen in the brain during SINV infection is largely mediated by an external force, such as by the host immune response. This is supported by previous studies that propose that the majority of neuronal death due to apoptosis and glutamate excitotoxicity is the work of T cells and astrocytes rather than being directly virus induced (45, 46).

Another surprising result was that disease severity did not correlate with increased viral titer or vRNA abundance. This is most clearly seen in the N376A-infected mice, where the viral titers and vRNA levels in the brains of mice that died are interspersed with those that survived. In addition, surviving mice from both the D355A and N376A infections had titers and vRNA levels in the brain that were roughly equivalent to those found in the mice infected with wild-type SINV that died. Furthermore, neither viral titer nor vRNA burden in the brain correlated with the levels of inflammation seen in Fig. 7. This suggests that, during SINV infection, high viral titer alone is not sufficient to cause severe disease in mice. In addition, altering capping efficiency did not significantly impact viral replication, dissemination, or tropism. This was illustrated by the roughly wild-type-equivalent titers found in the ankle, serum, and brain, indicating that both of the capping mutants were able to efficiently replicate at the site of inoculation, disseminate into the blood, and traffic to the brain. However, the slight decrease in N376A titer seen in the blood and brain does suggest that dissemination is slightly delayed or impaired when capping efficiency is decreased and may explain the slight increase seen in mean survival time.

Unfortunately, one question we were unable to answer in this study was whether viral capping efficiency in brain tissue was similarly affected by the nsP1 mutations, as was previously shown in tissue culture model systems and with recombinant proteins (21, 47). Regrettably, the limitations in sensitivity of previously established and currently available assays render us unable to directly answer this question, as these methods require a significant quantity of high-quality viral RNA that is difficult to obtain from brain tissue. This is likely due to the fact that an exceptionally small number of cells in the brain are required to be infected for the manifestation of significant disease and the appearance of endpoint criteria. However, the altered pathogenesis seen in mice in the absence of any obvious defects in viral replication, dissemination, or tropism lead us to believe that the point mutations incorporated are still altering capping efficiency and are not significantly affecting nsP functions in other ways. Previously characterized mutations in nsP1 that resulted in the loss of neurovirulence did so by significantly altering vRNA synthesis and/or processing of the nonstructural polyprotein, which typically resulted in decreased viral titer in animal models of infection (16, 17, 48, 49). Given that neither the D355A nor N376A mutation significantly altered viral titer or vRNA burden during SINV infection *in vivo* or negatively impacted vRNA synthesis or viral translation *in vitro*, we can conclude that the phenotypes seen both in tissue culture and in animal models of infection are the result of the mutations altering ncgRNA production through modulating capping efficiency. Furthermore, given the conserved effect of the D355A and N376A nsP1 point mutations in multiple alphaviruses in tissue culture and *in vitro*, it is likely that these mutations still increase or decrease, respectively, capping efficiency *in vivo*, but the magnitude by which capping efficiency is altered may be different from what was previously seen in tissue culture (21, 47).

### Noncapped genomic RNAs determine SINV virulence by modulating the host inflammatory response.

Infection with the D355A capping mutant resulted in the decreased expression of multiple cytokines and chemokines associated with the recruitment of immune cells, regulating inflammation, and apoptosis. While there was a small number of cytokines found to be differentially expressed during infection with the N376A mutant compared to wild-type SINV, they did not implicate any pathways in particular. Interestingly, expression of anti-inflammatory transcripts such as transforming growth factor beta (TGF-β) and IL-10 was found to be similar between D355A and wild-type infection, while others, such as SOCS1, were found to be significantly decreased. This suggests that the decreased inflammation seen with the D355A mutant is due to decreased activation of antiviral and inflammatory pathways rather than increased expression of anti-inflammatory cytokines. The decreased activation of these antiviral pathways are likely due to both the reduced release of DAMPS from dying cells and decreased sensing of viral PAMPS. The first is supported by the identification of several of the affected proteins being involved in apoptosis as well as the decreased level of cell death seen with the D355A mutant. The second is supported by the decreased IFN-β and ISG expression seen during D355A infection (Fig. 4). The decreased sensing of viral PAMPS may be due to the D355A mutant either being more efficient at inhibiting the cell’s viral sensors and signaling pathways through shutoff of host transcription or the D355A mutant producing fewer noncapped RNAs, which are established PAMPs (40, 41). Given that viral infection in animals is a continuous process, the shutoff of cellular transcription and suppression of the IFN response in tissues likely does not occur as efficiently or completely as it does in cell culture, where all the cells are infected simultaneously. Therefore, the decreased production of inflammatory cytokines seen with the D355A mutant is likely due to reduced detection of DAMPs and PAMPs caused by decreased cell death and decreased production of ncgRNAs. How exactly the ncgRNAs are sensed by the host during viral infection is not currently known and is an ongoing interest in the Sokoloski laboratory. While there is some evidence that suggests that the noncapped RNAs produced during alphaviral infection are at least in part sensed by RIG-I, it is unknown if this is also true for ncgRNAs, and there may be additional methods for detecting noncapped vRNAs that have yet to be characterized (41, 50). Overall, the correlation between decreased inflammation and decreased ncgRNA production leads us to conclude that the ncgRNAs play a critical role in determining the host response to viral infection.

In conclusion, we have identified a novel determinant of Sindbis virulence, which operates through a mechanism separate from those previously described. Specifically, decreasing the production of ncgRNAs by increasing capping efficiency results in the loss of neurovirulence, which we believe is due to the reduced production of RNA PAMPs that would otherwise cause excess inflammation and wide-spread cell death in the brain. The D355A mutation differs from previously identified nsP1 virulence determinants in that it does not negatively affect viral titer or resistance to IFN, such as is seen with the SINV nsP1 cleavage mutant T538I and the 6 nsP1 mutations characterized in Ross River virus (16, 19). The D355 residue in nsP1 is also unique from the aforementioned mutation sites in that it is very highly conserved among SINV strains as well as across both the old and new world alphaviruses. While the results of this paper indicate that the production of ncgRNAs is critical to SINV pathogenesis, more work is needed to further characterize the mechanisms by which ncgRNAs contribute to alphaviral neurovirulence.

## MATERIALS AND METHODS

### Tissue culture cells.

BHK-21 cells (a gift from R. W. Hardy, Indiana University–Bloomington) and L929 cells (a gift from P. Danthi, Indiana University–Bloomington) were maintained in minimal essential medium (MEM; Cellgro) containing fetal bovine serum (FBS; Atlanta Biologicals), 1% penicillin-streptomycin (Cellgro), 1% nonessential amino acids (Cellgro), and 1% l-glutamine (Cellgro). SK-N-BE(2) nerve cells (a gift from L. Beverly, University of Louisville) were maintained in Dulbecco's modified Eagle medium (DMEM)/F12 medium containing 10% FBS, 1% penicillin-streptomycin, and 1% l-glutamine. All cell lines were cultured at 37°C and 5% CO_2_ in a humidified incubator. Regular passaging using standard subculturing techniques was used to maintain low-passage-number stocks.

### Generation of AR86 SINV capping mutants.

The AR86 SINV nsP1 mutants used in this study were generated by Gibson Assembly via the use of a Gibson Assembly HiFi 1-step kit (SGI), using a restriction-digested AR86 cDNA plasmid and a synthetic DNA fragment, according to the manufacturer’s instructions (5). Mutants were verified by whole-genome sequencing; full-genome sequences are available upon request.

### Production of wild-type and mutant SINV stocks.

Wild-type, D355A, and N376A SINV AR86 were prepared by electroporation, as previously described (51). Approximately 2.8 × 10^6^ BHK-21 cells were electroporated with 10 μg of *in vitro*-transcribed RNA. This was done using a single pulse at 1.5 kV, 12 mA, and 200 Ω from a Gene Pulse Xcell system (Bio-Rad) as previously described (21). Afterwards, cells were incubated under normal conditions until cytopathic effect was apparent, at which point the supernatant was collected, clarified via centrifugation at 10,000 × *g* for 10 min at 4°C, and aliquoted into small-volume stocks, which were stored at −80°C for later use.

### Capping assay.

To define the impact of the nsP1 mutations on the capping activity of the alphaviral replicase complex, a linker-ligation-mediated approach was used. Briefly, BHK-21 cells were infected with the aforementioned SINV AR86 nsP1 mutants at a multiplicity of infection (MOI) of 5 infectious units (IU) per cell, and at 16 h postinfection (hpi), total RNA was extracted from the cells via TRIzol. As our previous studies have indicated that the 5′ end of the noncapped viral transcripts are polyphosphorylated, the total RNA samples must be initially dephosphorylated prior to completing the linker-ligation method (which requires a 5′ monophosphate) (20). To this end, 1 μg of RNA was dephosphorylated via treatment with Antarctic phosphatase (M0289S; NEB) per the manufacturer’s instructions. After a 30-min incubation period at 37°C, the Antarctic phosphatase was heat inactivated by incubating the reaction mixture for 2 min at 80°C prior to rapid cooling on ice. The dephosphorylated RNAs were then equally divided into two reaction mixtures to further prepare the noncapped and capped transcripts for linker-ligation and qRT-PCR analysis. To enable the detection of the noncapped viral RNAs, the dephosphorylated RNAs were treated with T4 polynucleotide kinase (PNK; M0201L; NEB) per the manufacturer’s instructions. In parallel, the capped RNAs were further prepared for linker-ligation via incubation in the presence of RNA 5′ pyrophosphohydrolase (RppH; M0356S; NEB) per the manufacturer’s instructions. Both the T4 PNK and RppH reaction mixtures were incubated for a period of 30 min at 37°C prior to phenol-chloroform extraction and ethanol precipitation. The RNA pellets were resuspended in 20 μl of nuclease-free water and used as the input materials for the linker-ligation reaction described below to enable the detection of the noncapped and capped vRNAs via qRT-PCR.

The prepared RNAs were then ligated to an RNA linker that was blocked on the 5′ end with a 9-carbon spacer (RNA linker, 5′-5Sp9-GUUCAGAGUUCUACAGUCCGACCCAUC-3′) via T4 RNA ligase 1. Briefly, each 30-μl reaction mixture consisted of 0.5 μg of prepared RNA (as described above), 1× T4 RNA ligase buffer, 1 mM ATP (final concentration), 1.66 μM RNA linker oligonucleotide (final concentration), 10 U of T4 RNA ligase 1 (M0204S; NEB), and 40 U of RNase inhibitor (M0314S; NEB). The linker-ligation reaction mixtures were incubated at 25°C for a period of 2 h prior to phenol-chloroform extraction and ethanol precipitation. The linker-ligated RNAs were resuspended in 20 μl of nuclease-free water, and 5 μl of the ligated RNAs was used as the input for reverse transcription (RT) reactions using OneScript plus RT (G237; Abmgood) per the manufacturer’s instructions. To enable the specific amplification of the viral genomic RNA, the RT reactions were primed with SINV.nSP1.R oligonucleotide (5′-AACATGAACTGGGTGGTGTCGAAG-3′). The composition of the SINV genomic RNA 5′ ends was then quantitatively assessed via qRT-PCR as previously described (20). The primers used for this experiment are listed in Table S1 in the supplemental material.

10.1128/mBio.02675-20.2 TABLE S1 Primers used in this study. Download Table S1, DOCX file, 0.01 MB. Copyright © 2020 LaPointe et al. 2020 LaPointe et al. This content is distributed under the terms of the Creative Commons Attribution 4.0 International license.

### Analysis of viral growth kinetics.

To determine if the mutation of the SINV nsP1 protein negatively impacted AR86 SINV infection, one-step viral growth kinetics for each capping mutant were assayed in tissue culture models of infection. BHK-21 cells were seeded in a 12-well plate and incubated under normal conditions until cell monolayers were 80 to 90% confluent. The cells were then infected with either wild-type virus or the individual capping mutant virus at an MOI of 5 IU/cell, and the virus was allowed to adsorb for 1 h. The inoculum was then removed, the cells were washed with 1× phosphate-buffered saline (PBS) to remove any unbound viral particles, and whole medium supplemented with 25 mM HEPES was added. The cells were incubated at 37°C, and tissue culture supernatants were harvested (and the medium replaced) at the indicated times postinfection. Viral titer was then determined via plaque assay.

### Quantification of infectious virus by plaque assay.

To determine the infectious viral titers of all viral samples produced during this study, standard virological plaque assays were used. To summarize, BHK-21 cells were seeded in 24-well plates under normal incubation conditions until the cell monolayers were 80 to 90% confluent. At that point, the cells were inoculated with 10-fold serial dilutions of virus-containing samples followed by a 1-h adsorption period. Afterwards, cells were overlaid with a solution of 0.5% Avicel (FMC Corporation) in 1× medium for 48 h (52). The monolayers were then fixed with formaldehyde solution (3.8% formaldehyde−1× PBS) for at least 1 h. The overlay was then removed, and the plaques were visualized via crystal violet staining.

### Western blotting.

To determine whether or not altering the capping efficiency impacted the expression of the SINV nonstructural genes, the expression of nsP2 was assessed via Western blotting. Briefly, whole-cell lysates were generated from BHK-21 cells that were infected with either wild-type SINV AR86, one of the above-described nsP1 mutants, or mock infected. At 8 h postinfection, the cells were lysed via the addition of radioimmunoprecipitation assay (RIPA) buffer (50 mM Tris-HCl [pH 7.5]–50 mM NaCl–1% [vol/vol] Nonidet P40 [NP-40]–0.5% [wt/vol] SDS–0.05% [wt/vol] sodium deoxycholate–1 mM EDTA) followed by vigorous vortexing prior to storage at −80°C until further use. Equal amounts of whole-cell lysates were resolved using SDS-PAGE and transferred to nitrocellulose membranes for downstream immunodetection. The resulting blots were probed for anti-SINV nsP2 polyclonal sera (a gift from R.W. Hardy at Indiana University−Bloomington) and anti-actin (clone mAGGEa; ThermoFisher) and probed with the appropriate horseradish peroxidase (HRP)-labeled secondary antibodies using the iBind Flex Western system with HRP detection/blotting reagents (ThermoFisher). Detection of the SINV nsP2 and host actin proteins was accomplished via chemiluminescence with SuperSignal West Pico Plus chemiluminescent substrate (34579; ThermoFisher) and detected by an Azure C200 Imaging Station (C200; Azure Biosystems).

### RNA kinetics.

BHK-21 cells were infected with either wild-type SINV AR86 or one of the aforementioned SINV nsP1 mutants at an MOI of 5 IU/cell. At the indicated times postinfection, the total RNA was isolated from the infected cells via TRIzol reagent according to the manufacturer’s instructions. Paired RT reactions were assembled using 1 μg of total cellular RNA and primer sets designed to prime the synthesis of cDNA from the viral RNA species in a transcript-specific manner. Briefly, the positive-sense RNAs were primed for cDNA synthesis using SINV.nsP1.R (5′-AACATGAACTGGGTGGTGTCGAAG-3′) and SINV.E1.R (5′-ATTGACCTTCGCGGTCGGATACAT-3′), and the negative-sense RNAs were primed for cDNA synthesis using SINV.nsP1.F (5′-AAGGATCTCCGGACCGTA-3′). All RT reactions also included an oligonucleotide priming for the mammalian 18S rRNA, Mam.18S.R (5′-AGTCGGCATCGTTTATGGTC-3′). qRT-PCR detection of the viral RNA species was accomplished using a standard curve analysis and subtractive method as previously described (21). Primer pairs are listed in Table S1.

### Type I IFN sensitivity assay.

L929 cells were seeded in a 48-well plate and, upon reaching 80 to 90% confluence, were inoculated with either wild-type parental virus or one of the individual capping mutants at an MOI of 10 IU/cell. After a 1-h adsorption period, the inoculum was removed, the cells were washed twice with 1× PBS, and whole medium was added. At the indicated times postinfection, 20 IU of murine type I IFN (R&D Systems) was added to the medium. Supernatants were collected at 24 hpi, and viral titer was determined by plaque assay.

### Detection of ISG and IFN-β transcripts.

To determine the abundance of IFN-β and the listed ISG transcripts, L929 cells were seeded in a 24-well plate and, upon reaching 80 to 90% confluence, were inoculated with wild-type SINV or one of the capping mutants at an MOI of 10 IU/cell. Additionally, L929 cells were mock infected with PBS to determine baseline IFN-β and ISG expression. After a 1-h adsorption period, the inoculum was removed, the cells were washed once with 1× PBS, and whole medium was added. At the specified time points, medium was removed, the cells were washed once with 1× PBS, cell lysates were harvested, and RNA was extracted using acidic guanidinium thiocyanate-phenol-chloroform extraction (53). The RNA was then DNase treated and precipitated via phenol-chloroform extraction. Following precipitation, 1 μg of RNA was reverse transcribed using random hexamer primer, and qRT-PCR was carried out as described above with primer sets obtained from PrimerBank. The sequences of these primers can be found in the supplemental material.

### Mouse experiments.

Four-week-old C57BL/6 mice were obtained from Jackson Laboratory and were inoculated in the left, rear footpad with 1,000 PFU of virus in diluent (1× PBS) in a volume of 10 μl. Mock-infected animals were injected with diluent alone. Mice were monitored for neurological signs of disease and weighed twice daily. The following neurological scoring was used: 0, no signs of overt disease and normal behavioral activity; 1, abnormal trunk curl, grip, or tail weakness (1 of 3); 2, abnormal trunk curl, grip, or tail weakness (2 of 3); 3, absent trunk curl, lack of gripping, tail paralysis; 4, pronounced dragging of one or more limbs; 5, hind or fore limb paralysis. On the termination day for each experiment or when mice met endpoint criteria (neurological score of 5 or 4 if the animal was unable to obtain food or water) or weight loss greater than 20% of initial body weight, the mice were sedated with isoflurane and euthanized by thoracotomy. Blood was then collected and serum obtained by collecting blood in serum separator tubes. Following exsanguination, tissues were collected by dissection. Tissues were then placed in 1× PBS and homogenized using Kimble BioMasher II closed-system micro tissue homogenizers. Ankle tissue was processed by bead beating using a Bead Ruptor 4 (Omni International). The infectious virus present in the tissue was quantified by plaque assay.

For histology, uninfected and SINV-infected mouse brains were removed at day 7 postinfection and were divided in half sagitally. One-half was used to assess viral titer (described above), while the remaining half was fixed in 4% formaldehyde and sectioned in paraffin. Tissue sections were then stained with hematoxylin and eosin (H&E). Pathological changes were scored by a board-certified veterinary pathologist (through the Comparative Pathology Core Services facility, Iowa State University) in the indicated categories, and regions of the brain were given the following scores: 0, normal; 1, minimal; 2, mild; 3, moderate; and 4, severe.

### Detection of viral genome and cytokine transcripts in mouse tissues.

To measure the level of viral genome and cytokine transcripts in tissues of infected mice, RNA was extracted from tissue homogenate using acidic guanidinium thiocyanate-phenol-chloroform extraction. The RNA was then DNase treated and precipitated via phenol-chloroform extraction. Following precipitation, 1 μg of RNA was reverse transcribed using Protoscript II reverse transcriptase (NEB) and random hexamer primer. The RNA genome was detected using BrightGreen Express qPCR master mix (Abmgood) and the following primer set specific for nsP1: F, 5′-AAGGATCTCCGGACCGTA-3′, and R, 5′-AACATGAACTGGGTGGTGTCGAAG-3′. A standard curve of known concentrations was used to determine the absolute quantities of viral genomic RNAs. Cytokine transcripts were detected using TaqMan Fast advanced master mix and the Applied Biosystems TaqMan array mouse immune response plates (catalog number 4414079) according to the manufacturer’s instructions.

### Neuron viability.

Neuron viability was determined using a previously described method of ethidium bromide and acridine orange staining (54). SK-N-BE(2) cells were seeded in a 96-well plate and, upon reaching 80 to 90% confluence, were inoculated with either wild-type SINV or one of the capping mutants at an MOI of 30 IU/cell. After a 1-h adsorption period, the inoculum was removed, the cells were washed once with 1× PBS, and DMEM/F12 medium was added. At 24 hpi, cell viability was assessed using ethidium bromide/acridine orange staining as described in Ribble et al. (54). Briefly, the 96-well plate was centrifuged at 1,000 rpm for 5 min using an Allegra 25R model centrifuge (Beckman Coulter) with inserts for 96-well plates. Following centrifugation, 8 μl of EB/AO dye solution (100 μg/ml ethidium bromide and 100 μg/ml acridine orange in 1× PBS) was added to each well. Cells were viewed using an epifluorescence microscope. Tests were done in triplicate, and a minimum of 100 total cells per well were counted using ImageJ.

### Animal ethics and research.

This study was carried out in strict accordance with the recommendations described in the *Guide for the Care and Use of Laboratory Animals* of the National Institutes of Health (55). The protocol was approved by the Institutional Animal Care and Use Committee of the University of Louisville (approval number 17-140). All manipulations that could result in acute pain or distress were performed under isoflurane anesthesia.

### Statistical analysis.

Unless otherwise stated, the quantitative data presented in this study represent the means of data from a minimum of three independent biological replicates. The *in vivo* studies described in this study were performed in duplicate using two independent preparations of viral stocks. An area under the curve approach was used to statistically assess the growth curve data presented in Fig. 1D to determine the differences in viral growth kinetics through the course of the assay. Comparative samples were statistically analyzed as previously described (51), using variable bootstrapping where appropriate. The survival data presented in Fig. 6 were statistically analyzed using the log rank test. Student's *t* test was used to determine the *P* values associated with individual quantitative data sets. Significance for data presented in Fig. 10 was determined using the Benjamini and Hochberg correction, and the corrected *P* values are shown in Fig. 10B.
