# Supplementary figures and images for: Production of Noncapped Genomic RNAs Is Critical to Sindbis Virus Disease and Pathogenicity
Source: mBio. 2020 Dec 1;11(6):e02675-20. doi: 10.1128/mBio.02675-20 (PMC7733944; doi:10.1128/mBio.02675-20)

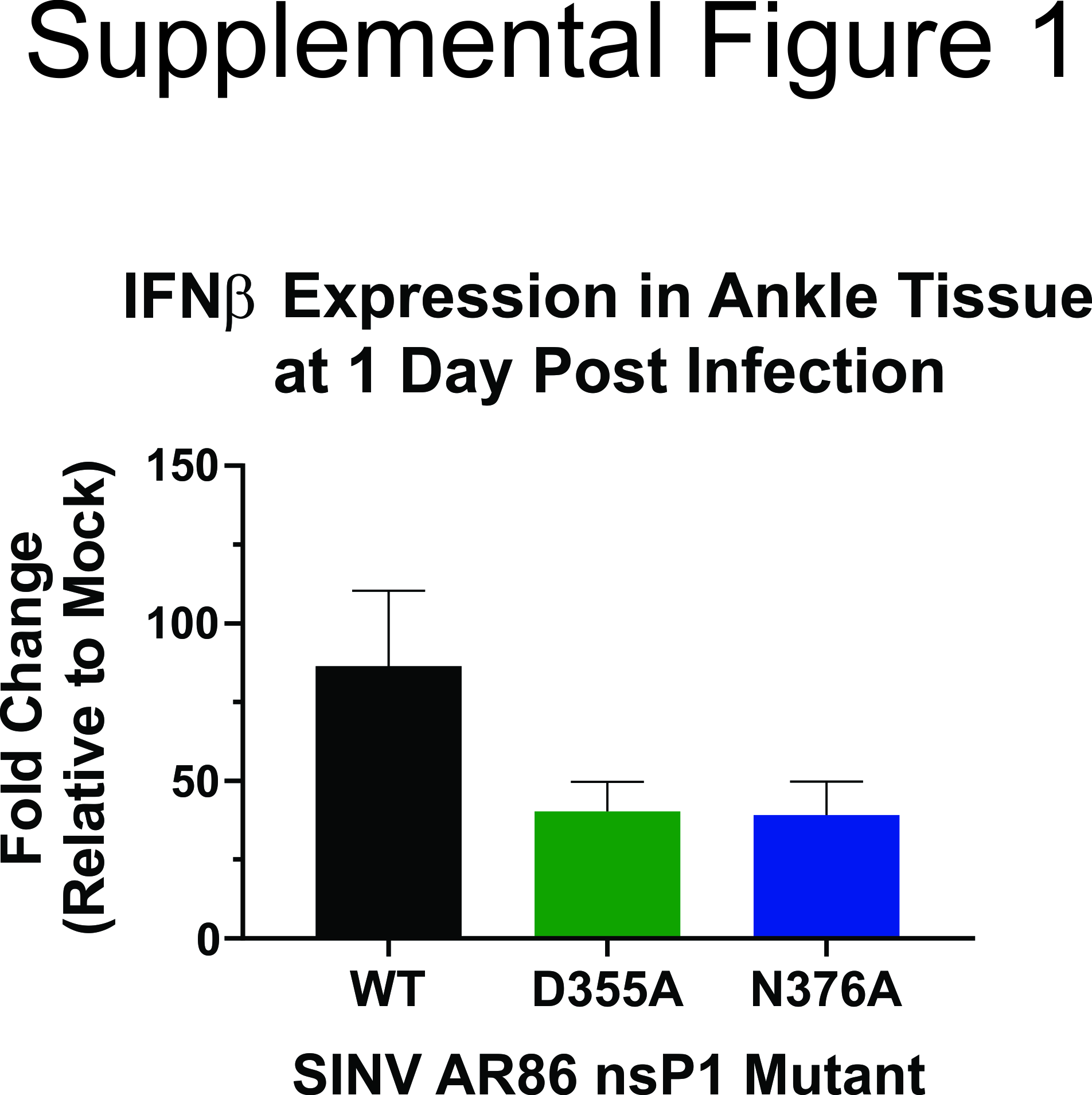

Supplement: FIG S1 [file mBio.02675-20-sf001.tif]
