# Supplementary material for: Production of Noncapped Genomic RNAs Is Critical to Sindbis Virus Disease and Pathogenicity
Source: mBio. 2020 Dec 1;11(6):e02675-20. doi: 10.1128/mBio.02675-20 (PMC7733944; doi:10.1128/mBio.02675-20)
Supplement: TABLE S1 [file mBio.02675-20-st001.docx]

**Supplemental Data Table 1-**

Linker= F: GTTCAGAGTTCTACAGTCCGACCCATC

SINVg.CapAssay= R: CGTCTACGTTTACTACTGGCTTCTCC

SINV.nsP1 =F: AAGGATCTCCGGACCGTA, R: AACATGAACTGGGTGGTGTCGAAG

SINV.E1= F: TCAGATGCACCACTGGTCTCAACA, R: ATTGACCTTCGCGGTCGGATACAT

Mam.18S= F: CGCGGTTCTATTTTGTTGGT, R: AGTCGGCATCGTTTATGGTC

IFIT2= F: AGTACAACGAGTAAGGAGTCACT, R: AGGCCAGTATGTTGCACATGG

SHB= F: CTGATGACTACTCCGATCCCTT, R: GGGGTGTCGTACAACTGGATG

CXCL10= F: CCAAGTGCTGCCGTCATTTTC, R: GGCTCGCAGGGATGATTTCAA

IFIH1= F: AGATCAACACCTGTGGTAACACC, R: CTCTAGGGCCTCCACGAACA

Viperin= F: TGCTGGCTGAGAATAGCATTAGG, R: GCTGAGTGCTGTTCCCATCT

MX2= F: GAGGCTCTTCAGAATGAGCAAA, R: CTCTGCGGTCAGTCTCTCT

OAS2= F: TTGAAGAGGAATACATGCGGAAG, R: GGGTCTGCATTACTGGCACTT

BST2= F: TGTTCGGGGTTACCTTAGTCA, R: GCAGGAGTTTGCCTGTGTCT

IFNβ= F: AAGAGTTACACTGCCTTTGCCATC, R: CACTGTCTGCTGGTGGAGTTCATC

GAPDH= F: AGGTCGGTGTGAACGGATTTG, R: TGTAGACCATGTAGTTGAGGTCA
